# Saliva and Plasma Reflect Metabolism Altered by Diabetes and Periodontitis

**DOI:** 10.3389/fmolb.2021.742002

**Published:** 2021-09-13

**Authors:** Akito Sakanaka, Masae Kuboniwa, Naoto Katakami, Masahiro Furuno, Hitoshi Nishizawa, Kazuo Omori, Naohiro Taya, Asuka Ishikawa, Shota Mayumi, Emiko Tanaka Isomura, Iichiro Shimomura, Eiichiro Fukusaki, Atsuo Amano

**Affiliations:** ^1^ Department of Preventive Dentistry, Osaka University Graduate School of Dentistry, Osaka, Japan; ^2^ Department of Metabolic Medicine, Osaka University Graduate School of Medicine, Osaka, Japan; ^3^ Department of Biotechnology, Osaka University Graduate School of Engineering, Osaka, Japan; ^4^ First Department of Oral and Maxillofacial Surgery, Osaka University Graduate School of Dentistry, Osaka, Japan

**Keywords:** metabolome, inflammation, biomarkers, saliva, non-alcoholic fatty liver disease

## Abstract

Periodontitis is an inflammatory disorder caused by disintegration of the balance between the periodontal microbiome and host response. While growing evidence suggests links between periodontitis and various metabolic disorders including type 2 diabetes (T2D), non-alcoholic liver disease, and cardiovascular disease (CVD), which often coexist in individuals with abdominal obesity, factors linking periodontal inflammation to common metabolic alterations remain to be fully elucidated. More detailed characterization of metabolomic profiles associated with multiple oral and cardiometabolic traits may provide better understanding of the complexity of oral-systemic crosstalk and its underlying mechanism. We performed comprehensive profiling of plasma and salivary metabolomes using untargeted gas chromatography/mass spectrometry to investigate multivariate covariation with clinical markers of oral and systemic health in 31 T2D patients with metabolic comorbidities and 30 control subjects. Orthogonal partial least squares (OPLS) results enabled more accurate characterization of associations among 11 oral and 25 systemic clinical outcomes, and 143 salivary and 78 plasma metabolites. In particular, metabolites that reflect cardiometabolic changes were identified in both plasma and saliva, with plasma and salivary ratios of (mannose + allose):1,5-anhydroglucitol achieving areas under the curve of 0.99 and 0.92, respectively, for T2D diagnosis. Additionally, OPLS analysis of periodontal inflamed surface area (PISA) as the numerical response variable revealed shared and unique responses of metabolomic and clinical markers to PISA between healthy and T2D groups. When combined with linear regression models, we found a significant correlation between PISA and multiple metabolites in both groups, including threonate, cadaverine and hydrocinnamate in saliva, as well as lactate and pentadecanoic acid in plasma, of which plasma lactate showed a predominant trend in the healthy group. Unique metabolites associated with PISA in the T2D group included plasma phosphate and salivary malate, while those in the healthy group included plasma gluconate and salivary adenosine. Remarkably, higher PISA was correlated with altered hepatic lipid metabolism in both groups, including higher levels of triglycerides, aspartate aminotransferase and alanine aminotransferase, leading to increased risk of cardiometabolic disease based on a score summarizing levels of CVD-related biomarkers. These findings revealed the potential utility of saliva for evaluating the risk of metabolic disorders without need for a blood test, and provide evidence that disrupted liver lipid metabolism may underlie the link between periodontitis and cardiometabolic disease.

## Introduction

Periodontitis is a chronic multifactorial inflammatory condition of the oral cavity caused by imbalanced interaction between the periodontal microbiome and host inflammatory response (Lamont et al., 2018). Although there are diverse clinical phenotypes (Papapanou et al., 2018), the disease affects the majority of adults worldwide, with a greater than 10% global prevalence rate of its severe form (Kassebaum et al., 2014), often characterized by destructive inflammation and tooth loss. In addition, this disease contributes to systemic chronic inflammatory disorders, such as obesity and related pathologies, including metabolic syndrome, type 2 diabetes (T2D), nonalcoholic fatty liver disease/nonalcoholic steatohepatitis (NAFLD/NASH), and cardiovascular disease (CVD) (Genco and Sanz, 2020). Although chronic low-grade inflammation is regarded as a unifying feature as well as contributor to linkage between periodontitis and these pathologies (Hajishengallis and Chavakis, 2021), the underlying mechanisms related to metabolic dysfunction remain to be fully elucidated. Moreover, such cardiometabolic diseases are closely intertwined, which has led to calls for caution when focusing solely on the association between periodontitis and a single cardiometabolic outcome, and also highlights the need for integrated analyses of multimodal molecular and clinical variables.

Metabolites, small molecules that reflect biological processes, are often measured in clinical medicine as diagnostic, prognostic, or treatment response biomarkers (Wishart, 2019). Recent advances in high-throughput technology have enabled systematic assessment of a metabolome, a collection of metabolites, in relation to cardiometabolic changes, and several studies that used combinations of blood metabolome and gut microbiome have characterized disease-associated metabolic pathways, including amino acid and fatty acid metabolism (Fan and Pedersen, 2021). Previously, we focused on the association of the salivary metabolome with periodontitis and showed its potential to reflect disease severity (Kuboniwa et al., 2016; Sakanaka et al., 2017). Nevertheless, there is a notable paucity of metabolomic approaches to address the complexity of oral-systemic crosstalk, such as how cardiometabolic phenotypes are reflected in saliva and how periodontal inflammation relates to systemic metabolic alterations.

The present study aimed to discover multivariate covariation patterns between metabolomes, as well as clinical features of periodontal and cardiometabolic traits, and then identify the most prominent features driving these associations. To achieve this, comprehensive profiling of plasma and salivary metabolomes was performed, and multivariate covariations with clinical markers of oral and systemic health in T2D patients and healthy subjects were investigated. To go beyond simple correlations, orthogonal partial least square (OPLS), a powerful multivariate method, was used. OPLS is tailored for analyses of high-dimensional datasets in which variables are expected to be correlated and does not require data reduction (Wheelock and Wheelock, 2013). Using results obtained with a combination of that method with periodontal inflamed surface area (PISA), a numerical representation of periodontitis severity (Nesse et al., 2008), the present report presents a catalog of salivary metabolites that potentially reflect cardiometabolic changes, and provides insight into the underlying link between periodontitis and cardiometabolic diseases.

## Materials and Methods

### Study Population

This study was approved by the Osaka University Research Ethics Committee, and performed in accordance with the principles of the Helsinki Declaration and STROBE guidelines for human observational studies. All participants provided written informed consent prior to enrollment and provided samples at Osaka University Medical Hospital. Systemically healthy controls and T2D patients, diagnosed using the criteria of the Japan Diabetes Society (Haneda et al., 2018), were recruited from November 2017 through March 2019. T2D patients were selected from individuals visiting the clinic of Metabolic Medicine at Osaka University Medical Hospital, and those experiencing severe renal dysfunction or end-stage renal failure (serum creatinine >2.0 mg/dl) were excluded. Healthy controls were recruited from volunteers employed by Osaka University or individuals visiting the clinic of Preventive Dentistry at Osaka University Dental Hospital. Exclusion criteria for controls included abnormal salivary function, use of antibiotics within the previous 3 months, use of prescription drugs within the previous 2 weeks, diagnosis of any disease in oral soft and hard tissues, or the presence of other systemic conditions.

### Blood and Urine Sample Collection, and Laboratory Measurements

All blood and urine samples were collected after an overnight fast, and used for blood and urine biochemical tests such as HbA1c and urine albumin based on standard laboratory protocols. At the same time, fasting plasma used for metabolomics was collected and kept at 4°C in a freezer (CubeCooler®; Forte Grow Medical, Tochigi, Japan), then subsequently frozen at −80°C. All participants were asked to complete various surveys in relation to demographics, current and past medical history, medications, smoking history, and family history, as well as anthropometry. Determination of hypertension (defined as systolic blood pressure ≥130 mmHg; diastolic blood pressure ≥80 mmHg; or anti-hypertensive medication use), dyslipidemia [defined as serum low-density lipoprotein cholesterol (LDL-C) ≥120 mg/dl; serum triglycerides (TG) ≥150 mg/dl; high-density lipoprotein cholesterol (HDL-C) <40 mg/dl; or lipid-lowering medication use], and obesity (BMI ≥25 kg/m^2^) were based on the criteria of the Japan Diabetes Society. For cardiometabolic disease risk scores, the participants were first categorized into quintiles of each blood biomarker level by ranking HbA1c, total cholesterol, TG, high-sensitivity C-reactive protein (hsCRP), and serum urate from lowest to highest with scores from 1 to 5. For HDL-C, the scoring was reversed. A cardiometabolic disease risk score was then calculated by summing these components, with a higher score indicating a higher risk of cardiometabolic disease.

### Oral Examination and Saliva Sample Collection

Four calibrated and licensed dentists performed oral examinations, and saliva collection the same day as blood and urine collection. Oral examinations include a full-mouth general dental survey and detailed periodontal assessment. All subjects were asked to refrain from eating, drinking, or brushing for at least 1 h prior to undergoing these procedures. Periodontal assessment included recording of probing depths, bleeding on probing, gingival recession, and clinical attachment level at six sites of all teeth present. Those results were used for calculating PISA (Nesse et al., 2008) and defining cases of periodontitis (Eke et al., 2012), the same as in our previous studies (Kuboniwa et al., 2016; Sakanaka et al., 2017). Plaque index (PlI) was determined as previously described (Sakanaka et al., 2017) or by scoring before dividing by number of teeth (sumPlI). The amount of tongue coating was scored as previously described (Oho et al., 2001). The subject was then asked to expectorate unstimulated whole saliva over a 10-min period into a 50-ml tube (Corning, Corning, NY, United States) kept on ice. For four diabetes patients and one healthy control with saliva output of less than 3 ml/10 min, they were asked to hold 3 ml of distilled water (HPLC grade; Sigma-Aldrich, St. Louis, MO, United States) in their mouth and then spit into a tube. We confirmed that there is no change in the main results when the samples with this treatment were excluded, by making the corrections described in the saliva and plasma metabolomics. Following incubation on ice for 15 min, the aqueous layer of each sample was pipetted off, and samples with volumes of at least 1 and 0.1 ml were aliquoted into 2-ml tubes kept at 4°C in a CubeCooler® as study and quality control (QC) samples, respectively. Subsequently, they were frozen with liquid nitrogen and stored at −80°C until analysis.

### Saliva and Plasma Metabolomics

Saliva samples were thawed at 4°C, then vortexed and centrifuged (4°C, 18,000 × g) for 3 min. Next, 0.8 ml of the aqueous layer was pipetted off and weighed, then 0.3 ml of that was transferred into a 2-ml glass vial (Nichiden-Rika Glass, Kobe, Japan) and kept at 4°C in a CubeCooler®. For extraction, 0.3 ml of deaerated Milli-Q water containing ribitol (0.02 mg/ml) as an internal standard was added. After incubation using an Eppendorf thermomixer (25°C, 1,000 rpm, 10 min), 1.4 ml of deaerated acetonitrile was added. After incubation (25°C, 1,000 rpm, 10 min) and centrifugation (4°C, 1800 × g) for 3 min, 1.6 ml of the supernatant was transferred to a 2-ml tube and dried with a vacuum concentrator (VC-96R; TAITEC, Koshigaya, Japan) for 30 min, then allowed to lyophilize overnight. Derivatization was performed with a methoxyamine hydrochloride solution with pyridine at a concentration of 20 mg/ml, followed by silylation application of N-methyl-N-(trimethylsilyl)-trifluoroacetamide (MSTFA). Gas chromatography coupled with mass spectrometry (GC/MS) analysis was performed on a GCMS-TQ8040 (Shimadzu, Kyoto, Japan) equipped with an AOC-20i autosampler (Shimadzu), a SKY™ liner (Restek, Bellefonte, PA, United States), and an InertCap 5MS/NP capillary column (0.25 mm × 30 m, 0.25 µm; GL Sciences, Tokyo, Japan), operated in full MS scan mode. Samples (1 µl each) were injected in split mode (split ratio 1:10). Helium gas flow rate through the column was set at 1.5 ml/min, with the column temperature set to 80°C for 2 min, then raised to 325°C over 15 min and held for 10 min. The temperature of the transfer interface was set to 310°C and of the ion source to 280°C. The selected mass range was set to 85–500 m/z with electron impact ionization. All saliva samples were prepared in random order and data were acquired in five batches. QC samples consisting of an equimolar mixture of all saliva samples and n-alkane mix C9-C40 (GL Sciences) containing decafluorotriphenylphosphine (DFTPP) (Sigma-Aldrich) were injected every five biological samples to monitor MS signal drift and perform locally weighted scatter plot smoothing (LOWESS) normalization for subsequent data processing. GC-MS data were converted into ABF format, then processed using MS-DIAL (version 3.90) to perform feature detection, spectra deconvolution, metabolite identification, and peak alignment (Tsugawa et al., 2015). Normalization was then performed based on the internal standard (ribitol) as well as the LOWESS algorithm, whereby metabolic feature signal drift with time was independently corrected by fitting a LOWESS curve to the MS signal measured in QCs. The acquired peak list was further normalized by sample weight (g/ml). Metabolic features from blanks and those with coefficient of variation in QCs above 30% were discarded. A total of 976 salivary metabolites were measured using our metabolite profiling platform, among which 143 metabolites were identified by matching retention time and fragmentation spectra to authentic standards, or by comparing fragmentation spectra to public repositories. Plasma samples were prepared and analyzed with a GC-MS/MS-TQ8040 in multiple reaction monitoring mode, as previously described (Katakami et al., 2020; Taya et al., 2021). Metabolomics data are available at Metabolomics Workbench (Study ID: ST001905 and ST001906).

### Statistical Analysis

An OPLS model of HbA1c was constructed using SIMCA-P software, v.16 (Umetrics, Umeå, Sweden) by setting HbA1c as the Y response variable, with all other parameters as X variables, which were all block-scaled by unit variance prior to analysis so that the influence of blocks of variables could be balanced in relation to their size. A seven-round cross-validation was performed to avoid model over-fitting. Quality and performance of the model was assessed by *R* ^2^ (goodness of fit) and *Q* ^2^ (goodness of prediction) values, cross-validation analysis of variance (CV-ANOVA), and a permutation test (assessment of risk of over-fitting). Variable relevance to explain the HbA1c variation was assessed using variable importance in projection on predictive component (VIP predictive) of the OPLS model as well as Spearman’s correlation, with *p* values corrected for multiple tests by controlling the false discovery rate using the Benjamini-Hochberg method. OPLS models of other clinical parameters were computed likewise, except for hypertension, for which OPLS discriminant analysis (OPLS-DA) was performed. For network analysis, the most relevant plasma and salivary metabolites against each parameter were selected by the combination of the p(corr) value (loading scaled as a correlation coefficient ranging from −1.0 to 1.0 between a model and original data) and the VIP predictive value from the OPLS models, as well as Spearman’s correlation. Networks were visualized using Gephi, v.0.9.2. Prediction performance was also evaluated by receiver operating characteristic (ROC) curve and area under the ROC curve (AUC). Associations were also tested by linear regression analysis. HbA1c models were adjusted for age, gender, smoking, waist circumference, HDL-C, TG, and PISA, HDL-C models for age, gender, smoking, waist circumference, HbA1c, TG, and PISA, and PISA models for age, gender, and smoking. A shared and unique structure (SUS) plot was formed with SIMCA to compare variable loadings of two OPLS models. When considered clinically relevant, specific variables were selected based on their position on the SUS plot [p(corr) value <−0.2 or >0.2]. Spearman’s correlation and Benjamini-Hochberg correction were performed using GraphPad Prism software, v.8. ROC curves and linear regression models were performed with the R package (v4.0.3).

## Results

**FIGURE 1:**
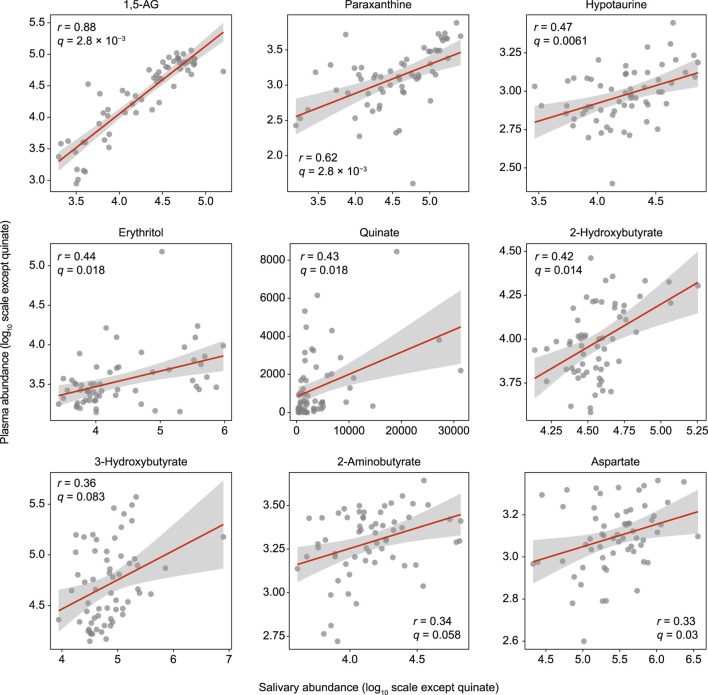
Significant associations of salivary metabolites with plasma counterparts. Spearman’s correlation analysis was used for 61 metabolites detected in both plasma and saliva. The Benjamini-Hochberg method was used to calculate *q* values (false discovery rate adjusted *p* value). Data are plotted on log scales, except for quinate, as that was not detected in some of the plasma samples.

The study population consisted of 30 systemically healthy controls and 31 T2D patients from an initial sample of 33, as two later refused to participate despite initial agreement (Supplementary Table S1). Using untargeted GC/MS, 143 salivary and 78 plasma metabolites were identified. Of these, 61 metabolites were found to be shared between them, nine of which showed a significant positive association (Spearman’s correlation value >0.3, *q* (adjusted *p* value) <0.1; Figure 1). Among those, 1,5-anhydroglucitol (1,5-AG) demonstrated the strongest positive association between plasma and saliva (*r* = 0.88, *q* = 2.8 × 10^–3^). Overall, a rich dataset comprised of four data blocks (11 oral and 25 systemic clinical outcomes, 143 salivary and 78 plasma metabolites) was generated.

**FIGURE 2:**
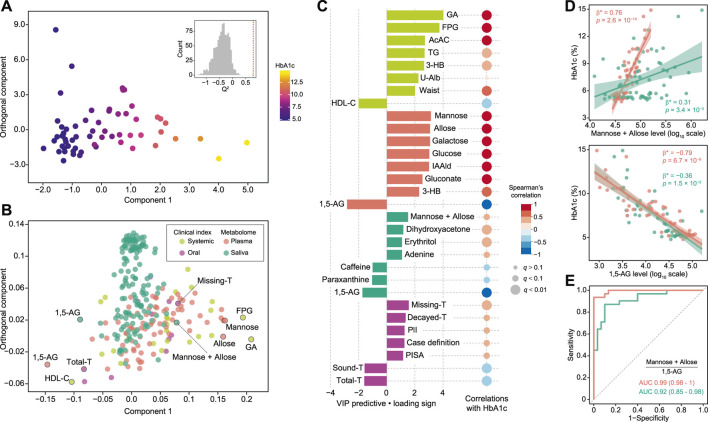
Global analysis of correlation of HbA1c with clinical and metabolomic features. **(A)** OPLS score plot showing distribution of study subjects according to HbA1c. Inset shows statistical validation using permutation analysis (*n* = 999 permutations, *Q* ^2^ = −0.4088 ± 0.268, data shown as mean ± SD). **(B)** OPLS loading plot showing color-coded distribution of predictors from four data blocks (pear: systemic clinical indices, purple: oral clinical indices, red: plasma metabolome, mint: saliva metabolome), with the right side associated with higher HbA1c. **(C)** Top predictors for HbA1c from each data block. A maximum of eight variables with importance for projection of a predictive component (VIP predictive) >1 were selected. The association was also assessed by Spearman’s correlation, with *p* values adjusted using the Benjamini-Hochberg method. **(D)** Significant associations of HbA1c with plasma (red) and salivary (mint) mannose and allose, or 1,5-anhydroglucitol (1,5-AG). Standardized β-estimates (β*) and *p* values were obtained using linear regression models, which were adjusted for age, gender, smoking, waist circumference (Waist), high-density lipoprotein cholesterol (HDL-C), triglyceride (TG), and PISA. **(E)** ROC curves comparing discriminative performance for T2D patients using plasma (red) and salivary (mint) ratios of (mannose + allose):1,5-AG. GA, glycated albumin; FPG, fasting plasma glucose; AcAC, acetoacetate; 3-HB, 3-hydroxybutyrate; U-Alb, urine albumin; IAAld, indoleacetaldehyde; T, number of teeth; PlI, plaque index.

For more accurate characterization of the associations between clinical and metabolomic measures, and to weigh the relative importance of variables in relation to HbA1c, OPLS was performed with HbA1c as a Y response variable. The model showed a good predictive ability of 0.788 for *Q* ^2^ and reliable performances during the permutation test (*n* = 999 permutations; Figure 2A, inset), as well as CV-ANOVA test (*p* value = 5.39 × 10^−15^, Supplementary Table S2). The loading plot indicated that a large portion of HbA1c variation was explained by data blocks of systemic clinical markers and plasma metabolome, including hyperglycemia and body fat markers, and circulating monosaccharides, which was also validated by Spearman’s correlation (Figures 2B,C; Supplementary Table S3). On the other hand, variations in salivary metabolome and oral clinical markers associated with HbA1c variation were relatively small. Looking at each block, the number of missing teeth ranked as the top predictor among oral clinical markers, followed by number of decayed teeth, plaque index, and definition as more severe cases of periodontitis and PISA, indicating that subjects with higher HbA1c had a more deteriorated oral health status with respect to both caries and periodontitis. Notably, substantial positive associations of HbA1c with both PISA and number of missing teeth, which normally have a tradeoff relationship, were noted, suggesting that the adverse effects of hyperglycemia on oral health are primarily manifested as destructive periodontal inflammation. Furthermore, several salivary metabolites were correlated with HbA1c. In particular, plasma and salivary levels of mannose and allose corresponded with high HbA1c levels, while those of 1,5-AG were found to correspond with low HbA1c levels (Figure 2C). Associations between these metabolites and HbA1c were significant, even after adjusting for multiple confounders (Figure 2D). Additionally, the plasma and salivary ratios of (mannose + allose):1,5-AG showed AUC values of 0.99 and 0.92, respectively, for T2D diagnosis (Figure 2E). These results suggest covariations among some circulating and salivary metabolites in response to hyperglycemia, highlighting the potential utility of saliva for diagnosis.

**FIGURE 3:**
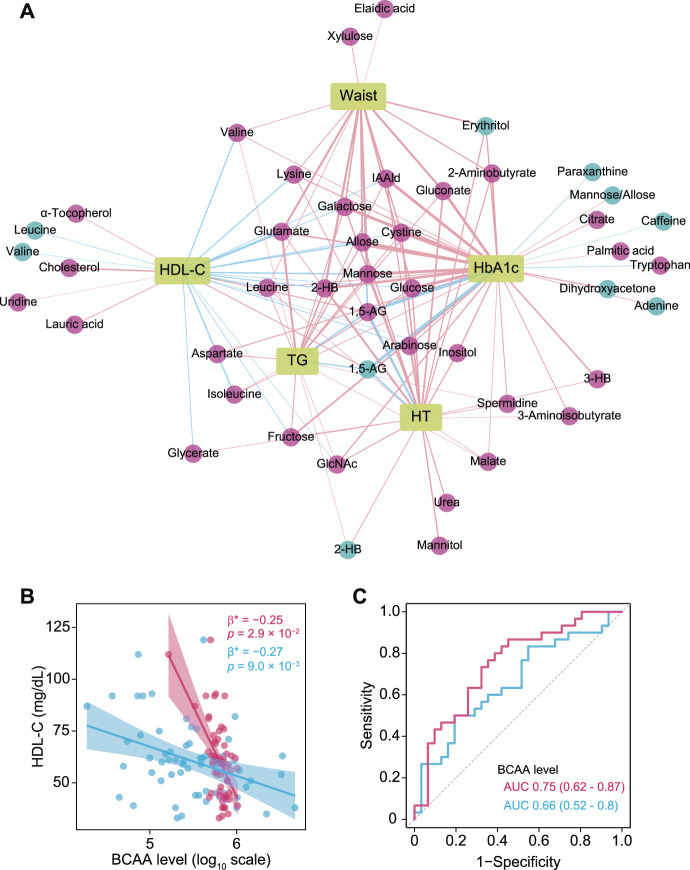
Associations of plasma and salivary metabolites with clinical indices of metabolic syndrome. **(A)** Correlation network of plasma and salivary metabolites associated with each pathology in metabolic syndrome that were selected by each OPLS model (VIP predictive value >1.0, p(corr) value <−0.3 or >0.3) as well as Spearman’s correlation coefficient <−0.3 and >0.3, and adjusted *p* value <0.1. Red and blue circles denote plasma and salivary metabolites, respectively. Red and blue edges denote positive and negative correlations, respectively. A thicker edge indicates a stronger correlation. **(B)** Significant associations of high-density lipoprotein cholesterol (HDL-C) with plasma (red) and salivary (blue) branched-chain amino acids (BCAA). Standardized β-estimates (β*) and *p* values were obtained from linear regression models, which were adjusted for age, gender, smoking, waist circumference (Waist), HbA1c, triglycerides (TG), and PISA. **(C)** ROC curves comparing the discriminative performance for HDL-C <60 mg/dl using plasma (red) and salivary (blue) levels of BCAAs. HT, hypertension; 1,5-AG, 1,5-anhydroglucitol; IAAld, indoleacetaldehyde; 2-HB, 2-hydroxybutyrate; 3-HB, 3-hydroxybutyrate.

To visualize correlations between clinical traits closely related to metabolic syndrome (HbA1c, HDL-C, TG, waist circumference, hypertension) and plasma and salivary metabolomes, OPLS models were computed against each clinical index (summarized in Supplementary Table S2), then the most relevant metabolites [VIP predictive value >1.0, p(corr) value <−0.3 or >0.3, Spearman’s correlation value <−0.3 or >0.3, *q* <0.1] were selected and a correlation network constructed. This analysis revealed common and unique metabolic signatures associated with each pathology related to metabolic syndrome (Figure 3A; Supplementary Table S4). Notably, dyslipidemia was characterized by multiple amino acids including branched-chain amino acids (BCAA) (Figure 3B), with plasma and salivary levels of BCAAs showing AUC values of 0.75 and 0.66, respectively, for HDL-C <60 mg/dl (Figure 3C).

**FIGURE 4:**
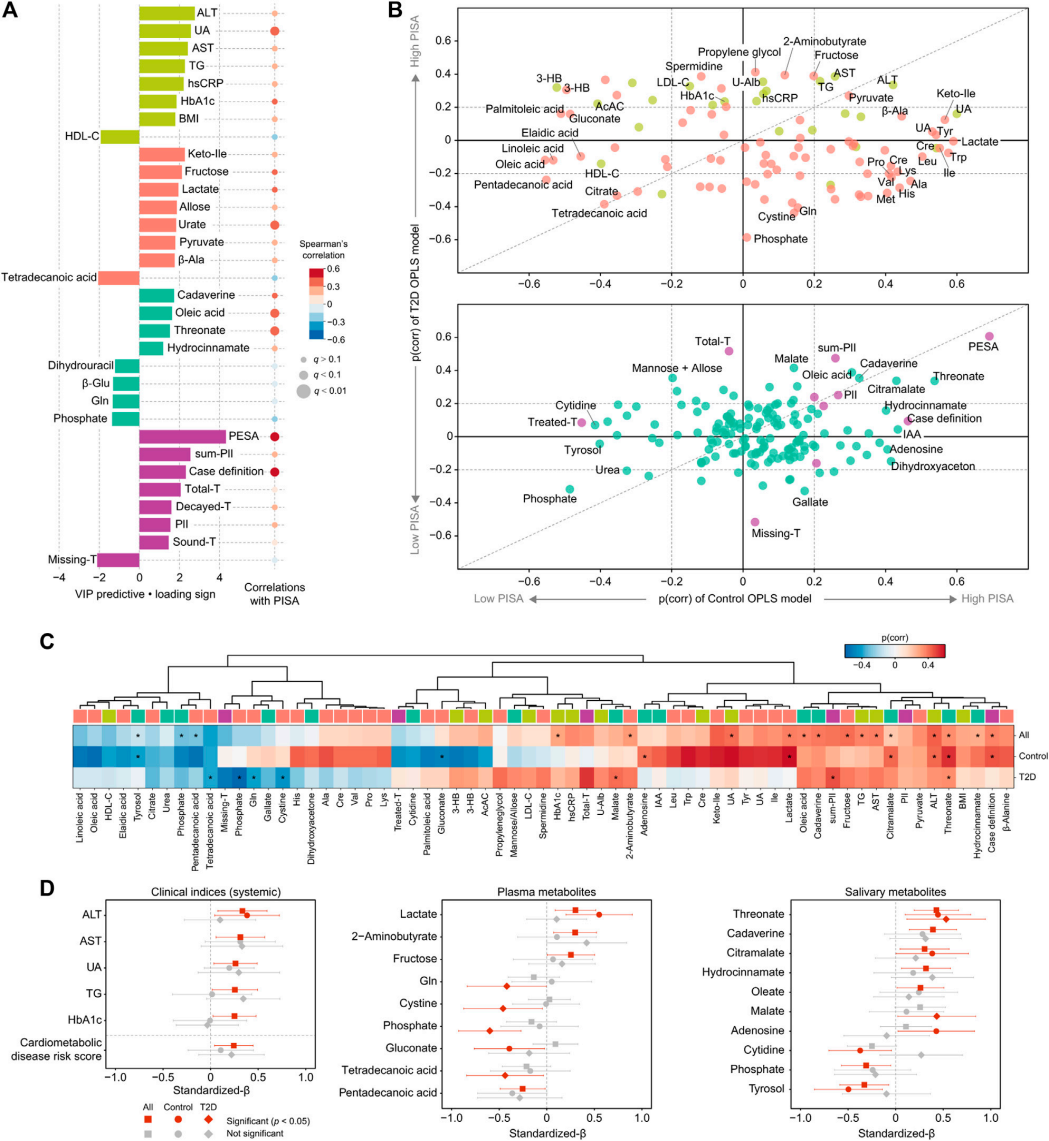
Global analysis of correlation of PISA with clinical and metabolomic features. **(A)** Top predictors for PISA from four data blocks (pear: systemic clinical indices, purple: oral clinical indices, red: plasma metabolome, mint: saliva metabolome) in OPLS analysis. A maximum of eight variables with importance for projection on a predictive component (VIP predictive) >1 were selected. The association was also assessed by Spearman’s correlation, with *p* values adjusted using the Benjamini-Hochberg method. **(B)** Shared and unique structures (SUS) plot constructed with p(corr) values from the two OPLS models of PISA using healthy subjects or T2D patients alone. This plot compares the influence of PISA on each variable between the healthy and T2D groups. Variables altered in both groups are clustered along the diagonal, while those located along the axes were specifically altered in the healthy or T2D group. **(C)** Heatmap of p(corr) values of clinical and metabolomic features impacted by PISA. Asterisks denote significant associations shown by linear regression analysis (*p* <0.05). **(D)** Standardized β-estimates with 95% confidence interval from linear regression analysis predicting PISA with each variable. All analyses were adjusted for age, gender, and smoking. Significant associations (*p* < 0.05) are highlighted by red. ALT, alanine aminotransferase; AST, aspartate aminotransferase; UA, urate; hsCRP, high-sensitivity C-reactive protein; Keto-Ile, ketoisoleucine; PESA, periodontal epithelial surface area; IAA, indoleacetic acid.

Next, the influence of periodontal inflammatory burden on cardiometabolic changes was assessed. OPLS analysis showed that a high PISA level was related to increased serum level of alanine aminotransferase (ALT), serum urate, aspartate aminotransferase (AST), TG, hsCRP and HbA1c, as well as other metabolites in plasma and saliva, including salivary cadaverine, oleic acid, threonate and hydrocinnamate (Figure 4A; Supplementary Table S5). To examine differences in PISA influence on each variable between the healthy and T2D groups, two OPLS models of PISA were constructed using healthy and T2D subjects alone, and an SUS plot was generated. As a result, ALT, AST, and TG were plotted together on the diagonal, which revealed a positive association with PISA in both groups (Figure 4Bi). Meanwhile, HbA1c and hsCRP showed a positive association with PISA only in the T2D group, while serum urate showed that in the healthy controls (Figure 4Bi). Gluconate and several fatty acids in plasma were clustered on the left, and lactate and several amino acids on the right, indicating negative and positive associations with PISA in the healthy group, respectively. Meanwhile, phosphate, cystine and glutamine in plasma were clustered on the bottom, and 2-aminobutyrate and fructose in plasma on the top, indicating negative and positive associations with PISA in the T2D group, respectively (Figure 4Bi). Positive associations between PISA and salivary levels of cadaverine, oleic acid, and citramalate were also noted in both groups, while salivary phosphate was plotted on the contrast diagonal (Figure 4Bii). Hydrocinnamate, adenosine, cytidine and tyrosol in saliva correlated with PISA only in the healthy controls, while salivary malate showed a positive association with PISA only in the T2D group (Figure 4Bii).

To validate these results, linear regression models adjusted for age, gender, and smoking were employed (Figures 4C,D). Positive associations with PISA were confirmed for ALT, AST, urate, TG, and HbA1c, while a number of aforementioned plasma and salivary metabolites also showed significant associations with PISA in all, or only the healthy or T2D group. For instance, we found a significant correlation between PISA and multiple metabolites in both groups, including threonate, cadaverine and hydrocinnamate in saliva, as well as lactate and pentadecanoic acid in plasma, of which plasma lactate showed a predominant trend in the healthy group. Unique metabolites associated with PISA in the T2D group included plasma phosphate and salivary malate, while those in the healthy group included plasma gluconate and salivary adenosine. Furthermore, a significant positive association of PISA with risk of developing CVD was noted based on a composite score summarizing HbA1c, total cholesterol, TG, HDL-C, hsCRP, and urate (Figure 4D, left bottom). Taken together, these results indicate that PISA has stronger associations with markers of hepatic-parenchymal damage as well as abnormal lipid metabolism among cardiometabolic profiles, suggesting that these abnormalities may underlie the link between periodontal inflammation and cardiometabolic changes.

## Discussion

This study demonstrated multivariate patterns of association among metabolome and clinical measures related to periodontal and cardiometabolic health in T2D patients, and highlights the potential utility of saliva for non-invasive assessment of risk to systemic health. Additionally, periodontal inflammation was shown to primarily affect several clinical markers including ALT, AST and TG, as well as plasma metabolomic profiles, which provides mechanistic insight into etiological links between periodontitis and cardiometabolic diseases.

Results of OPLS analysis with HbA1c as the outcome revealed that plasma and salivary levels of 1,5-AG, as well as mannose and allose exhibit concordant changes in response to HbA1c. Previous studies established plasma mannose and 1,5-AG as reliable T2D markers in addition to glucose, with plasma mannose implicated in insulin resistance (Lee et al., 2016), and incident T2D and CVD (Mardinoglu et al., 2017), while 1,5-AG was as a marker of short-term glycemic control (McGill et al., 2004). A positive association between plasma and salivary 1,5-AG has also been recently shown (Mook-Kanamori et al., 2014b; Jian et al., 2020). This is the first known report showing a substantial and significant covariation between plasma and salivary mannose, as well as findings demonstrating their diagnostic utility for hyperglycemia when combined with salivary 1,5-AG. We also found salivary erythritol to be positively correlated with HbA1c, in line with previous studies showing positive associations between T2D and levels of erythritol in saliva (Barnes et al., 2014) and plasma (Rebholz et al., 2018). Based on our results showing stronger associations between HbA1c and these salivary carbohydrates as compared to glucose, which is more likely to be consumed by numerous oral microbes, salivary reflection of the circulating metabolome may be influenced by oral microbe metabolic activities, which should be taken into account in future salivary diagnostics. Other salivary metabolites showing negative correlations with T2D include caffeine and paraxanthine, metabolites related to coffee consumption, implicated in lower risk of CVD (Ding et al., 2014). Although dietary habits were not evaluated in the present subjects, a relationship of those with T2D pathogenesis was suggested.

An inverse correlation of HDL-C with BCAAs was also found in both plasma and saliva, and unaffected after adjustment for multiple confounders including PISA. Elevated circulating BCAAs via gut dysbiosis is known as a strong biomarker for insulin resistance and increased risk of T2D (Wang et al., 2011; Pedersen et al., 2016). It has been shown that insulin resistance is accompanied by altered lipoprotein metabolism leading to higher TG and lower HDL-C (Ginsberg and Huang, 2000), and that an elevated level of circulating BCAAs is associated with higher TG and lower HDL-C in individuals with and without T2D (Mook-Kanamori et al., 2014a; Fukushima et al., 2019). Our results extend those findings to suggest that salivary BCAAs may be useful for reflecting plasma BCAAs as part of monitoring prediabetic markers. Meanwhile, periodontitis has been implicated in dyslipidemia and insulin resistance (Orlandi et al., 2020), and a recent animal study showed that the periodontal pathogen *Porphyromonas gingivalis* might aggravate insulin resistance together with elevated circulating BCAAs through its metabolic activity (Tian et al., 2020). The present study also found a negative though not significant association of PISA with HDL-C, and a positive association with plasma and salivary BCAAs, which warrants further investigation of the intricate relationship among periodontitis, dyslipidemia, and BCAAs. In addition to BCAAs, our correlation network analysis replicated a number of already known plasma metabolites related to T2D and other metabolic disorders, including glutamate, 2-hydroxybutyrate, indole acetaldehyde, and α-tocopherol (Fan and Pedersen 2021; Krautkramer et al., 2021), which appears to corroborate the validity of our measurements and analysis.

Another key finding is that PISA is directly proportional to hepatic-parenchymal damage markers such as ALT and AST. The present results add to growing evidence implicating periodontitis in the pathogenesis of NAFLD/NASH (Acharya et al., 2017). A recent cohort study presented findings indicating a correlation of periodontitis with incident liver disease independent of confounders (Helenius-Hietala et al., 2019). Furthermore, another report showed that periodontal treatment of NAFLD/NASH patients decreased ALT and AST levels, while intravenous administration of *P. gingivalis* significantly increased body and liver weights, and elevated ALT and TG in a mouse model of NAFLD induced by a high fat diet (Yoneda et al., 2012), consistent with our observations. Given the essential roles of the liver in both glucose and lipid metabolism, its functional impairment appears to a key explanatory factor behind the link between periodontitis and cardiometabolic diseases.

The present SUS-plot results of healthy subjects and T2D patients showed distinctive roles of PISA. An association of PISA with HbA1c and hsCRP was only seen in the patients, indicating that those with T2D are more susceptible to the effects of periodontal inflammation on glucose intolerance and systemic inflammation, and suggests the clinical importance of periodontal treatment for disease management, consistent with a previous report (Genco and Sanz, 2020). Additionally, our results showed a positive association between PISA and serum urate only in the healthy group. That is in line with results showing that hyperglycemia promotes urate excretion (Lipkowitz, 2012), as well as other studies that found elevated levels of circulating urate in subjects with periodontitis (Banu et al., 2015; Babaei et al., 2018). The differences in association between PISA and these clinical markers between healthy and T2D groups shown in this study may be important in considering the relationship between periodontitis, diabetes and cardiometabolic diseases.

We also noted an inverse association of PISA with a variety of circulating fatty acids, mostly in the healthy group. Although there are no known reports implicating these metabolites in periodontitis development, higher circulating levels of pentadecanoic and palmitoleic acid have been found to be associated with lower risk of cardiometabolic disease (Forouhi et al., 2014; Çimen et al., 2016; Kamleh et al., 2018). Although additional studies are needed, these results suggest that metabolic alterations in plasma fatty acids might be among factors underlying the link between periodontitis and cardiometabolic disease. The present study also found a positive correlation between PISA and plasma lactate only in the healthy group, which has also never been reported. Plasma lactate is known to be elevated in high-intensity exercise and critical illnesses such as sepsis and malignancy, and has recently been recognized as a new metabolic health marker rather than a traditional waste product (Brooks, 2018; Broskey et al., 2020). Although lactate is a common metabolite produced by oral bacteria, no significant association was found between PISA and salivary lactate in this study, so the increase in plasma lactate in periodontitis patients cannot be immediately linked to metabolism by oral microbes. In the future, it will be necessary to verify this relationship from both host and microbial perspectives.

As for salivary metabolites in relation to PISA, cadaverine and hydrocinnamate were shown to characterize the severity of periodontal disease in both healthy and T2D groups. This is in agreement with our previous studies (Kuboniwa et al., 2016; Sakanaka et al., 2017), which have also shown that these metabolites are likely to be of microbial origin by comparing metabolite profiles before and after plaque removal. Recent larger clinical studies have also shown that cadaverine, hydrocinnamate and their derivatives in saliva can be biomarkers of periodontitis or tooth loss resulting from it (Liebsch et al., 2019; Andörfer et al., 2021). The present study provides further evidence to support the predictive ability of these salivary metabolites against periodontal disease. Except for cadaverine and hydrocinnamate, the present results revealed a number of known metabolites related to periodontal health or disease such as phosphate (Liebsch et al., 2019), oleic acid (Barnes et al., 2011; Barnes et al., 2014) and citramalate (Pei et al., 2020), though also showed an additional novel group, which requires further validation.

The present study has some limitations and corresponding future research directions should be acknowledged. Although we aimed to characterize global covariation between oral and cardiometabolic phenotypes by including multiple parameters obtained through more detailed and accurate measurements, the cross-sectional design did not allow for establishment of causal relationships, while the relatively small sample size potentially limits extrapolation of the findings. Future investigations should seek to replicate and extend the present findings with a larger number of samples using a longitudinal approach.

In conclusion, our study provides not only a catalog of salivary metabolites that potentially reflect cardiometabolic changes but also findings linking periodontal inflammation to disrupted liver function and lipid metabolism. It is considered that these results provide a new starting point for further investigations of oral-systemic crosstalk. Given that saliva testing, which has received increased attention due to the coronavirus pandemic, will be more prevalent in the future, analysis of panels of salivary metabolites may not only become an attractive alternative to blood testing for screening of metabolic disorders, but may also help to reduce the daily burden on patients who need to manage their conditions over a long period of time. In addition, saliva testing during regular dental visits may allow early detection of metabolic disorders in people without subjective symptoms, thus making dentistry a potential gatekeeper for medical diseases.

## Data Availability

The original contributions presented in the study are included in the article/Supplementary Files, further inquiries can be directed to the corresponding author.

## Glossary

1,5-AG: 1,5-Anhydroglucitol
2-HB: 2-Hydroxybutyrate
3-HB: 3-Hydroxybutyrate
AcAC: Serum acetoacetate
Ala: Alanine
ALT: Alanine aminotransferase
AST: Aspartate aminotransferase
β-Ala: β-Alanine
β-Glu: β-Glutamate
BCAA: Branched-chain amino acid
BMI: Body mass index
Cre: Serum creatinine
Decayed-T: Number of decayed teeth
FPG: Fasting plasma glucose
GA: Glycated albumin
Gln: Glutamine
HDL-C: High-density lipoprotein cholesterol
His: Histidine
hsCRP: High-sensitivity C-reactive protein
HT: Hypertension
IAA: Indoleacetic acid
IAAld: Indoleacetaldehyde
Ile: Isoleucine
Keto-Ile: Ketoisoleucine
LDL-C: Low-density lipoprotein cholesterol
Leu: Leucine
Lys: Lysine
Missing-T: Number of missing teeth
PESA: Periodontal epithelial surface area
PISA: Periodontal inflamed surface area
PlI: Plaque index
Pro: Proline
Sound-T: Number of sound teeth
TG: Triglyceride
Total-T: Number of total teeth
Trp: Tryptophan
Tyr: Tyrosine
UA: Urate
U-Alb: Urine albumin
Val: Valine
