# Supplementary material for: Saliva and Plasma Reflect Metabolism Altered by Diabetes and Periodontitis
Source: Front Mol Biosci. 2021 Sep 13;8:742002. doi: 10.3389/fmolb.2021.742002 (PMC8473679; doi:10.3389/fmolb.2021.742002)
Supplement: Supplementary file 1 [file DataSheet1.docx]

**
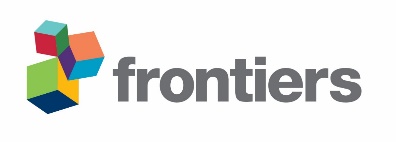
*Supplementary Material for***

**Saliva and Plasma Reflect Metabolism Altered by Diabetes and Periodontitis**

Akito Sakanaka^1^, Masae Kuboniwa^1^*, Naoto Katakami^2^, Masahiro Furuno^3^, Hitoshi Nishizawa^2^, Kazuo Omori^2^, Naohiro Taya^2^, Asuka Ishikawa^1^, Shota Mayumi^1^, Emiko Tanaka Isomura^4^, Iichiro Shimomura^2^, Eiichiro Fukusaki^3^, Atsuo Amano^1^

^1^Department of Preventive Dentistry, Osaka University Graduate School of Dentistry; 1-8 Yamadaoka, Suita, Osaka 565-0871, Japan

^2^Department of Metabolic Medicine, Osaka University Graduate School of Medicine; 2-2 Yamadaoka, Suita, Osaka 565-0871, Japan

^3^Department of Biotechnology, Osaka University Graduate School of Engineering; 2-1 Yamadaoka, Suita, Osaka 565-0871, Japan

^4^First Department of Oral and Maxillofacial Surgery, Osaka University Graduate School of Dentistry; 1-8 Yamadaoka, Suita, Osaka 565-0871, Japan

*Address correspondence to:

Masae Kuboniwa, DDS, PhD

Associate Professor

Department of Preventive Dentistry, Osaka University Graduate School of Dentistry

1-8 Yamadaoka, Suita, Osaka 565-0871, Japan

Tel: +81-6-6879-2922, Fax: +81-6-6876-2925

Email: kuboniwa@dent.osaka-u.ac.jp

**Supplementary Table 1.** Clinical characteristics of study subjects

|  | Total subjects | T2D subjects | Control subjects |
| --- | --- | --- | --- |
| *n* | 61 | 31 | 30 |
| Age (years)* | 53.16 (15.38) | 63.87 (10.50) | 42.10 (11.24) |
| Females, *n* (%) | 27 (44.3) | 18 (58.1) | 9 (30.0) |
| BMI (kg/m^2^)* | 24.83 (5.00) | 27.04 (5.11) | 22.55 (3.75) |
| <25, *n* (%) | 34 (55.7) | 10 (32.3) | 25 (83.3) |
| ≥25 to <30, *n* (%) | 20 (32.8) | 16 (51.6) | 3 (10.0) |
| ≥30, *n* (%) | 7 (11.5) | 5 (16.1) | 2 (6.7) |
| Waist circumference (cm)* | 90.26 (13.40) | 98.68 (10.36) | 81.56 (10.31) |
| Fasting plasma glucose (mg/dL)* | 118.16 (43.36) | 145.35 (46.09) | 90.06 (9.23) |
| HbA1c (%)* | 7.30 (2.39) | 9.14 (2.06) | 5.40 (0.32) |
| Glycated albumin (%)* | 18.82 (6.51) | 23.71 (5.73) | 13.77 (1.39) |
| Serum acetoacetate (μmol/L)* | 46.90 (60.94) | 84.67 (66.33) | 7.86 (5.52) |
| Serum 3-hydroxybutyrate (μmol/L)* | 105.32 (106.42) | 152.80 (128.90) | 56.26 (36.69) |
| Fasting serum insulin (μU/mL)* | 7.21 (4.85) | 5.97 (4.51) | 8.49 (4.94) |
| hsCRP (mg/dL)* | 0.12 (0.27) | 0.18 (0.34) | 0.066 (0.13) |
| Triglycerides (mg/dL)* | 120.24 (84.10) | 156.90 (97.33) | 82.36 (43.76) |
| HDL-C (mg/dL)* | 60.70 (18.46) | 52.70 (11.86) | 68.96 (20.51) |
| LDL-C (mg/dL)* | 122.49 (42.51) | 120.03 (48.52) | 125.03 (35.93) |
| Total cholesterol (mg/dL)* | 205.86 (51.59) | 200.67 (60.88) | 211.23 (40.18) |
| Dyslipidemia, *n* (%) | 26 (42.6) | 26 (83.9) | 0 (0) |
| Hypertension, *n* (%) | 22 (36.1) | 22 (71.0) | 0 (0) |
| AST (IU/L)* | 25.04 (12.90) | 27.87 (17.08) | 22.13 (5.00) |
| ALT (IU/L)* | 27.22 (21.28) | 30.64 (27.44) | 23.7 (11.51) |
| γ-GTP (IU/L)* | 33.39 (25.52) | 39.12 (30.36) | 27.46 (17.96) |
| CPK (IU/L)* | 100.24 (37.32) | 95.70 (37.29) | 104.93 (37.39) |
| Serum creatinine (mg/dL)* | 0.76 (0.20) | 0.78 (0.25) | 0.74 (0.13) |
| Serum uric acid (mg/dL)* | 5.61 (1.17) | 5.66 (1.15) | 5.56 (1.20) |
| Urine albumin (mg/L)* | 68.66 (263.73) | 120.78 (364.07) | 14.79 (29.65) |
| Urine creatinine (mg/dL)* | 114.25 (71.49) | 93.32 (53.75) | 135.89 (81.44) |
| Urine uric acid (mg/dL)* | 47.71 (27.68) | 38.59 (21.66) | 57.15 (30.31) |
| eGFR (mL/min/1.73m^2^)* | 78.19 (18.27) | 70.78 (21.49) | 85.85 (9.65) |
| Smoking status |  |  |  |
| Never, *n* (%) | 42 (68.9) | 16 (51.6) | 26 (86.7) |
| Former, *n* (%) | 18 (29.5) | 14 (45.2) | 4 (13.3) |
| Current, *n* (%) | 1 (1.6) | 1 (3.2) | 0 (0) |
| Brinkmann index* | 156.89 (330.78) | 287.51 (423.65) | 21.91 (60.27) |
| Number of teeth |  |  |  |
| Total* | 23.96 (5.64) | 21.25 (6.44) | 26.76 (2.66) |
| Decayed* | 1.47 (2.72) | 2.19 (3.14) | 0.73 (1.99) |
| Missing* | 4.13 (5.55) | 6.74 (6.44) | 1.43 (2.47) |
| Treated* | 7.91 (5.61) | 8.96 (5.75) | 6.83 (5.35) |
| Sound* | 14.31 (8.30) | 10.09 (7.43) | 18.66 (6.85) |
| Plaque index (PlI)* | 0.91 (0.48) | 1.05 (0.53) | 0.77 (0.39) |
| sumPlI* | 85.55 (47.16) | 88.64 (52.52) | 82.36 (41.56) |
| Salivary flow rate (mL/min) | 0.88 (0.46) | 0.79 (0.37) | 0.97 (0.53) |
| Tongue coating* (Oho et al. 2001) | 1.75 (1.39) | 2.03 (1.44) | 1.46 (1.30) |
| PESA* | 1182.08 (372.51) | 1107.54 (425.06) | 1259.09 (296.84) |
| PISA* | 350.62 (286.91) | 412.46 (242.99) | 286.73 (317.68) |
| Case definition* (Eke et al. 2012) |  |  |  |
| 0: No periodontitis, *n* (%) | 7 (11.5) | 0 (0) | 7 (23.3) |
| 1: Mild periodontitis, *n* (%) | 3 (4.9) | 0 (0) | 3 (10.0) |
| 2: Moderate periodontitis, *n* (%) | 38 (62.3) | 23 (74.2) | 15 (50.0) |
| 3: Severe periodontitis, *n* (%) | 13 (21.3) | 8 (25.8) | 5 (16.7) |
| Mean value* | 1.93 (0.85) | 2.25 (0.44) | 1.6 (1.03) |

Values are presented as the mean (SD), unless otherwise indicated.

*Parameters employed for multivariate analysis.

ALT, Alanine aminotransferase; AST, Aspartate aminotransferase; BMI, Body mass index; CPK, Creatine phosphokinase; eGFR, Estimated glomerular filtration rate; γ-GTP, γ-Glutamyl transpeptidase; HDL-C, High-density lipoprotein cholesterol; hsCRP, High-sensitivity C-reactive protein; LDL-C, Low-density lipoprotein cholesterol; PESA, Periodontal epithelial surface area; PISA, Periodontal inflamed surface area; T2D, Type 2 diabetes.

**Supplementary Table 2.** Quality parameters for OPLS models

| Y response variable | Component, *n* | R^2^ | Q^2^ | CV-ANOVA *P* value |
| --- | --- | --- | --- | --- |
| HbA1c | 1 + 3 | 0.976 | 0.788 | 5.39 × 10^-15^ |
| HDL-C | 1 + 2 | 0.825 | 0.499 | 8.64 × 10^-7^ |
| Waist circumference | 1 + 6 | 0.995 | 0.627 | 4.71 × 10^-6^ |
| Triglycerides | 1 + 3 | 0.922 | 0.310 | 9.19 × 10^-3^ |
| Hypertension* | 1 + 1 | 0.660 | 0.306 | 3.43 × 10^-4^ |
| PISA | 1 + 1 | 0.687 | 0.220 | 7.54 × 10^-3^ |

Number of components depicted as numbers of predictive + orthogonal components.

*OPLS-discriminant analysis (OPLS-DA) was constructed.

CV-ANOVA, Cross-validation analysis of variance; HDL-C, High-density lipoprotein cholesterol; OPLS, Orthogonal partial least square; PISA, Periodontal inflamed surface area.

**Supplementary Table 3.** Clinical and metabolic predictors for HbA1c

| Variable | Class | OPLS | | Spearman’s | |
| --- | --- | --- | --- | --- | --- |
|  |  | p(corr) | VIP pred | rho | FDR |
| Glycated albumin | Systemic | 0.912894 | 4.02148 | 0.9085 | 0.00092 |
| Fasting plasma glucose | Systemic | 0.849369 | 3.74165 | 0.7791 | 0.00092 |
| Mannose | Plasma | 0.870806 | 3.13965 | 0.7993 | 0.00092 |
| Allose | Plasma | 0.854083 | 3.07935 | 0.8354 | 0.00092 |
| Galactose | Plasma | 0.854016 | 3.07911 | 0.789 | 0.00092 |
| Glucose | Plasma | 0.838786 | 3.0242 | 0.7865 | 0.00092 |
| Indoleacetaldehyde | Plasma | 0.835441 | 3.01214 | 0.7796 | 0.00092 |
| Acetoacetate | Systemic | 0.61617 | 2.71436 | 0.7579 | 0.00092 |
| Triglycerides | Systemic | 0.611498 | 2.69377 | 0.4711 | 0.00095 |
| 3-Hydroxybutyrate | Systemic | 0.604705 | 2.66385 | 0.4886 | 0.00092 |
| Gluconate | Plasma | 0.704609 | 2.54043 | 0.7682 | 0.00092 |
| 3-Hydroxybutyrate | Plasma | 0.63927 | 2.30486 | 0.5245 | 0.00092 |
| Urine albumin | Systemic | 0.511655 | 2.25395 | 0.208 | 0.22057 |
| 2-Hydroxybutyrate | Plasma | 0.592069 | 2.13467 | 0.7248 | 0.00092 |
| 2-Aminobutyrate | Plasma | 0.564031 | 2.03359 | 0.5782 | 0.00092 |
| Waist circumference | Systemic | 0.45727 | 2.01437 | 0.5324 | 0.00092 |
| Brinkmann index | Systemic | 0.443209 | 1.95243 | 0.4406 | 0.00301 |
| Fructose | Plasma | 0.540962 | 1.95041 | 0.5579 | 0.00092 |
| Inositol | Plasma | 0.536834 | 1.93553 | 0.4806 | 0.00092 |
| Age | Systemic | 0.429154 | 1.89051 | 0.5571 | 0.00092 |
| Glutamate | Plasma | 0.47758 | 1.72189 | 0.5247 | 0.00092 |
| Spermidine | Plasma | 0.469528 | 1.69286 | 0.4177 | 0.00554 |
| *N*-Acetylglucosamine | Plasma | 0.461275 | 1.6631 | 0.4806 | 0.00092 |
| Cystine | Plasma | 0.452262 | 1.63061 | 0.6311 | 0.00092 |
| 3-Aminoisobutyrate | Plasma | 0.43414 | 1.56527 | 0.4075 | 0.00741 |
| Number of missing teeth | Oral | 0.355222 | 1.56482 | 0.4744 | 0.00095 |
| Aspartate | Plasma | 0.431312 | 1.55507 | 0.4419 | 0.00301 |
| Mannose + Allose | Saliva | 0.489602 | 1.51702 | 0.3109 | 0.05085 |
| BMI | Systemic | 0.336876 | 1.48401 | 0.4065 | 0.00768 |
| Lysine | Plasma | 0.390357 | 1.40741 | 0.4489 | 0.0024 |
| Isoleucine | Plasma | 0.380945 | 1.37348 | 0.3764 | 0.01593 |
| Arabinose | Plasma | 0.373881 | 1.34801 | 0.4479 | 0.0024 |
| Number of decayed teeth | Oral | 0.304519 | 1.34147 | 0.3545 | 0.02331 |
| Plaque index | Oral | 0.287037 | 1.26446 | 0.3222 | 0.04231 |
| Malate | Plasma | 0.349852 | 1.26137 | 0.3633 | 0.02048 |
| Case definition | Oral | 0.28496 | 1.25531 | 0.3459 | 0.02781 |
| ALT | Systemic | 0.274593 | 1.20964 | 0.1552 | 0.38367 |
| hsCRP | Systemic | 0.273324 | 1.20405 | 0.4046 | 0.00768 |
| γ-GTP | Systemic | 0.270211 | 1.19033 | 0.2295 | 0.17189 |
| AST | Systemic | 0.267485 | 1.17833 | 0.1412 | 0.44156 |
| LDL-C | Systemic | 0.266669 | 1.17473 | 0.005482 | 0.98194 |
| Dihydroxyacetone | Saliva | 0.378985 | 1.17428 | 0.4655 | 0.00177 |
| Leucine | Plasma | 0.325109 | 1.17216 | 0.3857 | 0.0128 |
| PISA | Oral | 0.264029 | 1.1631 | 0.3327 | 0.03576 |
| Citrate | Plasma | 0.322456 | 1.1626 | 0.387 | 0.0128 |
| Palmitic acid | Plasma | 0.310554 | 1.11969 | 0.327 | 0.03918 |
| Valine | Plasma | 0.303866 | 1.09557 | 0.3715 | 0.01707 |
| Erythritol | Saliva | 0.34991 | 1.08419 | 0.489 | 0.00092 |
| Adenine | Saliva | 0.348979 | 1.08131 | 0.3659 | 0.01933 |
| Oleic acid | Plasma | 0.286824 | 1.03413 | 0.3296 | 0.03742 |
| Total cholesterol | Systemic | 0.230607 | 1.01587 | -0.03759 | 0.89658 |
| Caffeine | Saliva | -0.32364 | 1.0028 | -0.3157 | 0.04629 |
| CPK | Systemic | -0.23486 | 1.03462 | -0.2147 | 0.20781 |
| Paraxanthine | Saliva | -0.33671 | 1.0433 | -0.3502 | 0.0256 |
| Tryptophan | Plasma | -0.36613 | 1.32005 | -0.3565 | 0.02276 |
| Number of sound teeth | Oral | -0.35832 | 1.57847 | -0.4539 | 0.00177 |
| Number of total teeth | Oral | -0.36268 | 1.59767 | -0.4865 | 0.00092 |
| 1,5-AG | Saliva | -0.5577 | 1.72802 | -0.7891 | 0.00092 |
| HDL-C | Systemic | -0.45616 | 2.00949 | -0.4309 | 0.00366 |
| 1,5-AG | Plasma | -0.78379 | 2.82593 | -0.869 | 0.00092 |

ALT, Alanine aminotransferase; 1,5-AG, 1,5-Anhydroglucitol; AST, Aspartate aminotransferase; BMI, Body mass index; CPK, Creatine phosphokinase; FDR, False discovery rate; γ-GTP, γ-Glutamyl transpeptidase; HDL-C, High-density lipoprotein cholesterol; hsCRP, High-sensitivity C-reactive protein; LDL-C, Low-density lipoprotein cholesterol; Oral, Oral clinical indices; PISA, Periodontal inflamed surface area; Plasma, Plasma metabolites; Saliva, Salivary metabolites; Systemic, Systemic clinical indices; VIP pred, Variable importance in projection on predictive component.

**Supplementary Table 4.** Metabolites associated with clinical indices of metabolic syndrome

| Phenotype | Metabolite | Class | OPLS | | Spearman’s | |
| --- | --- | --- | --- | --- | --- | --- |
|  |  |  | p(corr) | VIP pred | rho | FDR |
| HbA1c | Mannose | Plasma | 0.870806 | 3.13965 | 0.7993 | 0.00092 |
| HbA1c | Allose | Plasma | 0.854083 | 3.07935 | 0.8354 | 0.00092 |
| HbA1c | Galactose | Plasma | 0.854016 | 3.07911 | 0.789 | 0.00092 |
| HbA1c | Glucose | Plasma | 0.838786 | 3.0242 | 0.7865 | 0.00092 |
| HbA1c | Indoleacetaldehyde | Plasma | 0.835441 | 3.01214 | 0.7796 | 0.00092 |
| HbA1c | Gluconate | Plasma | 0.704609 | 2.54043 | 0.7682 | 0.00092 |
| HbA1c | 3-Hydroxybutyrate | Plasma | 0.63927 | 2.30486 | 0.5245 | 0.00092 |
| HbA1c | 2-Hydroxybutyrate | Plasma | 0.592069 | 2.13467 | 0.7248 | 0.00092 |
| HbA1c | 2-Aminobutyrate | Plasma | 0.564031 | 2.03359 | 0.5782 | 0.00092 |
| HbA1c | Fructose | Plasma | 0.540962 | 1.95041 | 0.5579 | 0.00092 |
| HbA1c | Inositol | Plasma | 0.536834 | 1.93553 | 0.4806 | 0.00092 |
| HbA1c | Mannose + allose | Saliva | 0.489602 | 1.51702 | 0.3109 | 0.05085 |
| HbA1c | Glutamate | Plasma | 0.47758 | 1.72189 | 0.5247 | 0.00092 |
| HbA1c | Spermidine | Plasma | 0.469528 | 1.69286 | 0.4177 | 0.00554 |
| HbA1c | *N*-Acetylglucosamine | Plasma | 0.461275 | 1.6631 | 0.4806 | 0.00092 |
| HbA1c | Cystine | Plasma | 0.452262 | 1.63061 | 0.6311 | 0.00092 |
| HbA1c | 3-Aminoisobutyrate | Plasma | 0.43414 | 1.56527 | 0.4075 | 0.00741 |
| HbA1c | Aspartate | Plasma | 0.431312 | 1.55507 | 0.4419 | 0.00301 |
| HbA1c | Lysine | Plasma | 0.390357 | 1.40741 | 0.4489 | 0.0024 |
| HbA1c | Isoleucine | Plasma | 0.380945 | 1.37348 | 0.3764 | 0.01593 |
| HbA1c | Dihydroxyacetone | Saliva | 0.378985 | 1.17428 | 0.4655 | 0.00177 |
| HbA1c | Arabinose | Plasma | 0.373881 | 1.34801 | 0.4479 | 0.0024 |
| HbA1c | Erythritol | Saliva | 0.34991 | 1.08419 | 0.489 | 0.00092 |
| HbA1c | Malate | Plasma | 0.349852 | 1.26137 | 0.3633 | 0.02048 |
| HbA1c | Adenine | Saliva | 0.348979 | 1.08131 | 0.3659 | 0.01933 |
| HbA1c | Leucine | Plasma | 0.325109 | 1.17216 | 0.3857 | 0.0128 |
| HbA1c | Citrate | Plasma | 0.322456 | 1.1626 | 0.387 | 0.0128 |
| HbA1c | Palmitic acid | Plasma | 0.310554 | 1.11969 | 0.327 | 0.03918 |
| HbA1c | Valine | Plasma | 0.303866 | 1.09557 | 0.3715 | 0.01707 |
| HbA1c | Caffeine | Saliva | -0.32364 | 1.0028 | -0.3157 | 0.04629 |
| HbA1c | Paraxanthine | Saliva | -0.33671 | 1.0433 | -0.3502 | 0.0256 |
| HbA1c | Tryptophan | Plasma | -0.36613 | 1.32005 | -0.3565 | 0.02276 |
| HbA1c | 1,5-AG | Saliva | -0.5577 | 1.72802 | -0.7891 | 0.00092 |
| HbA1c | 1,5-AG | Plasma | -0.78379 | 2.82593 | -0.869 | 0.00092 |
| HDL-C | Cholesterol | Plasma | 0.58273 | 2.35015 | 0.5225 | 0.0023 |
| HDL-C | 1,5-AG | Plasma | 0.447214 | 1.80361 | 0.4328 | 0.00711 |
| HDL-C | Lauric acid | Plasma | 0.396469 | 1.59896 | 0.3884 | 0.02048 |
| HDL-C | Uridine | Plasma | 0.383877 | 1.54817 | 0.304 | 0.09572 |
| HDL-C | 1,5-AG | Saliva | 0.301241 | 1.04407 | 0.4268 | 0.00768 |
| HDL-C | α-Tocopherol | Plasma | 0.300459 | 1.21175 | 0.3609 | 0.03336 |
| HDL-C | Valine | Saliva | -0.30977 | 1.07362 | -0.3261 | 0.06431 |
| HDL-C | Inositol | Plasma | -0.34815 | 1.40408 | -0.3302 | 0.06016 |
| HDL-C | Fructose | Plasma | -0.3598 | 1.45106 | -0.3654 | 0.03243 |
| HDL-C | Leucine | Saliva | -0.36834 | 1.27662 | -0.3397 | 0.05258 |
| HDL-C | Cystine | Plasma | -0.3689 | 1.48776 | -0.3627 | 0.03336 |
| HDL-C | Galactose | Plasma | -0.37619 | 1.51716 | -0.3801 | 0.0237 |
| HDL-C | Glucose | Plasma | -0.38048 | 1.53449 | -0.3605 | 0.03336 |
| HDL-C | Indoleacetaldehyde | Plasma | -0.38487 | 1.55216 | -0.3827 | 0.02265 |
| HDL-C | Arabinose | Plasma | -0.38569 | 1.55548 | -0.3889 | 0.02048 |
| HDL-C | *N*-Acetylglucosamine | Plasma | -0.40146 | 1.6191 | -0.3308 | 0.06016 |
| HDL-C | Mannose | Plasma | -0.40757 | 1.64371 | -0.5065 | 0.0023 |
| HDL-C | Glycerate | Plasma | -0.41045 | 1.65534 | -0.3894 | 0.02048 |
| HDL-C | Allose | Plasma | -0.41403 | 1.66977 | -0.5031 | 0.0023 |
| HDL-C | 2-Hydroxybutyrate | Plasma | -0.44444 | 1.79243 | -0.4394 | 0.0064 |
| HDL-C | Lysine | Plasma | -0.47565 | 1.91829 | -0.4506 | 0.00549 |
| HDL-C | Valine | Plasma | -0.58704 | 2.36751 | -0.4645 | 0.00394 |
| HDL-C | Leucine | Plasma | -0.62111 | 2.50494 | -0.4632 | 0.00394 |
| HDL-C | Aspartate | Plasma | -0.62579 | 2.52379 | -0.4951 | 0.0023 |
| HDL-C | Isoleucine | Plasma | -0.65174 | 2.62848 | -0.4782 | 0.0023 |
| HDL-C | Glutamate | Plasma | -0.72158 | 2.91013 | -0.6839 | 0.0023 |
| Waist | Glutamate | Plasma | 0.58552 | 2.591 | 0.6304 | 0.00121 |
| Waist | 2-Hydroxybutyrate | Plasma | 0.525105 | 2.32365 | 0.6466 | 0.00121 |
| Waist | Mannose | Plasma | 0.515263 | 2.2801 | 0.5997 | 0.00121 |
| Waist | Allose | Plasma | 0.5069 | 2.24309 | 0.5827 | 0.00121 |
| Waist | Glucose | Plasma | 0.495896 | 2.1944 | 0.523 | 0.00121 |
| Waist | Cystine | Plasma | 0.457962 | 2.02654 | 0.5055 | 0.00121 |
| Waist | Indoleacetaldehyde | Plasma | 0.454921 | 2.01308 | 0.4941 | 0.00121 |
| Waist | Galactose | Plasma | 0.432449 | 1.91364 | 0.5191 | 0.00121 |
| Waist | 2-Aminobutyrate | Plasma | 0.430437 | 1.90473 | 0.4651 | 0.00223 |
| Waist | Gluconate | Plasma | 0.37901 | 1.67717 | 0.6414 | 0.00121 |
| Waist | Leucine | Plasma | 0.377952 | 1.67248 | 0.3661 | 0.0296 |
| Waist | Elaidic aicd | Plasma | 0.372144 | 1.64678 | 0.3152 | 0.0873 |
| Waist | Valine | Plasma | 0.365475 | 1.61727 | 0.368 | 0.0296 |
| Waist | Lysine | Plasma | 0.353103 | 1.56252 | 0.4038 | 0.01189 |
| Waist | Xylulose | Plasma | 0.320282 | 1.41728 | 0.3933 | 0.01501 |
| Waist | Erythritol | Saliva | 0.309771 | 1.17803 | 0.5315 | 0.00121 |
| Waist | 1,5-AG | Plasma | -0.51759 | 2.29039 | -0.5357 | 0.00121 |
| TG | Glutamate | Plasma | 0.58313 | 2.59388 | 0.6386 | 0.00197 |
| TG | Mannose | Plasma | 0.562618 | 2.50264 | 0.4897 | 0.00197 |
| TG | 2-Hydroxybutyrate | Plasma | 0.554996 | 2.46874 | 0.5516 | 0.00197 |
| TG | Allose | Plasma | 0.521258 | 2.31866 | 0.476 | 0.00197 |
| TG | Galactose | Plasma | 0.514255 | 2.28751 | 0.4199 | 0.01138 |
| TG | Indoleacetaldehyde | Plasma | 0.501928 | 2.23268 | 0.4077 | 0.01482 |
| TG | Glucose | Plasma | 0.499687 | 2.22271 | 0.4208 | 0.0112 |
| TG | 2-Hydroxybutyrate | Saliva | 0.468015 | 1.7891 | 0.3169 | 0.08856 |
| TG | Gluconate | Plasma | 0.463308 | 2.06089 | 0.4173 | 0.01138 |
| TG | Leucine | Plasma | 0.42561 | 1.8932 | 0.3913 | 0.02095 |
| TG | Fructose | Plasma | 0.409809 | 1.82291 | 0.4228 | 0.0112 |
| TG | Aspartate | Plasma | 0.398956 | 1.77464 | 0.4049 | 0.01536 |
| TG | Spermidine | Plasma | 0.390264 | 1.73597 | 0.3201 | 0.08604 |
| TG | Isoleucine | Plasma | 0.38899 | 1.73031 | 0.3413 | 0.06059 |
| TG | Valine | Plasma | 0.368375 | 1.63861 | 0.3284 | 0.0784 |
| TG | Cystine | Plasma | 0.354425 | 1.57655 | 0.4005 | 0.01707 |
| TG | *N*-Acetylglucosamine | Plasma | 0.35161 | 1.56404 | 0.3244 | 0.08378 |
| TG | Malate | Plasma | 0.335754 | 1.4935 | 0.3206 | 0.08604 |
| TG | 1,5-AG | Saliva | -0.41698 | 1.594 | -0.4768 | 0.00197 |
| TG | 1,5-AG | Plasma | -0.5194 | 2.3104 | -0.5108 | 0.00197 |
| HT | Glucose | Plasma | 0.73906 | 2.43729 | 0.4994 | 0.00143 |
| HT | 2-Hydroxybutyrate | Plasma | 0.711094 | 2.34506 | 0.6194 | 0.00143 |
| HT | Cystine | Plasma | 0.710665 | 2.34364 | 0.5584 | 0.00143 |
| HT | Mannose | Plasma | 0.703458 | 2.31988 | 0.547 | 0.00143 |
| HT | Inositol | Plasma | 0.698088 | 2.30217 | 0.4651 | 0.00214 |
| HT | Indoleacetaldehyde | Plasma | 0.695687 | 2.29425 | 0.4631 | 0.00214 |
| HT | Allose | Plasma | 0.684774 | 2.25826 | 0.5127 | 0.00143 |
| HT | Galactose | Plasma | 0.684647 | 2.25784 | 0.4994 | 0.00143 |
| HT | Gluconate | Plasma | 0.675557 | 2.22786 | 0.5604 | 0.00143 |
| HT | *N*-Acetylglucosamine | Plasma | 0.621306 | 2.04895 | 0.4346 | 0.00459 |
| HT | 3-Hydroxybutyrate | Plasma | 0.618306 | 2.03906 | 0.3602 | 0.02908 |
| HT | 2-Aminobutyrate | Plasma | 0.610355 | 2.01284 | 0.446 | 0.00297 |
| HT | Arabinose | Plasma | 0.580846 | 1.91552 | 0.4555 | 0.00214 |
| HT | Glutamate | Plasma | 0.560845 | 1.84956 | 0.3907 | 0.01512 |
| HT | Lysine | Plasma | 0.548964 | 1.81038 | 0.4003 | 0.01199 |
| HT | Urea | Plasma | 0.528287 | 1.74219 | 0.4612 | 0.00214 |
| HT | 3-Aminoisobutyrate | Plasma | 0.526733 | 1.73707 | 0.324 | 0.05705 |
| HT | Leucine | Plasma | 0.506665 | 1.67089 | 0.3488 | 0.03888 |
| HT | Spermidine | Plasma | 0.464135 | 1.53063 | 0.3755 | 0.0207 |
| HT | Glycerate | Plasma | 0.427717 | 1.41053 | 0.345 | 0.04176 |
| HT | Fructose | Plasma | 0.415932 | 1.37167 | 0.3888 | 0.01512 |
| HT | 2-Hydroxybutyrate | Saliva | 0.410254 | 1.1627 | 0.3888 | 0.01512 |
| HT | Erythritol | Saliva | 0.394348 | 1.11762 | 0.4612 | 0.00214 |
| HT | Mannitol | Plasma | 0.32851 | 1.08337 | 0.4975 | 0.00143 |
| HT | Malate | Plasma | 0.303482 | 1.00083 | 0.3316 | 0.05257 |
| HT | 1,5-AG | Saliva | -0.55909 | 1.58452 | -0.5222 | 0.00143 |
| HT | 1,5-AG | Plasma | -0.8015 | 2.64318 | -0.6194 | 0.00143 |

1,5-AG, 1,5-Anhydroglucitol; FDR, False discovery rate; HDL-C, High-density lipoprotein cholesterol; HT, Hypertension; Plasma, Plasma metabolites; Saliva, Salivary metabolites; TG, Triglycerides; VIP pred, Variable importance in projection on predictive component; Waist, Waist circumference.

**Supplementary Table 5.** Clinical and metabolic predictors for PISA

| Variable | Class | OPLS (All) | | OPLS (T2D) | | OPLS (Control) | |
| --- | --- | --- | --- | --- | --- | --- | --- |
|  |  | p(corr) | VIP pred | p(corr) | VIP pred | p(corr) | VIP pred |
| PESA | Oral | 0.685912 | 4.31607 | 0.606337 | 3.54459 | 0.691281 | 3.28884 |
| ALT | Systemic | 0.439239 | 2.76389 | 0.335315 | 1.96022 | 0.421034 | 2.00311 |
| Serum uric acid | Systemic | 0.408307 | 2.56926 | 0.160181 | 0.9364 | 0.599894 | 2.85405 |
| sumPlI | Oral | 0.40453 | 2.54549 | 0.474112 | 2.77161 | 0.259272 | 1.23351 |
| AST | Systemic | 0.383827 | 2.41521 | 0.386663 | 2.26039 | 0.258002 | 1.22747 |
| Case definition | Oral | 0.367615 | 2.3132 | 0.094436 | 0.552066 | 0.462918 | 2.20238 |
| Triglycerides | Systemic | 0.36111 | 2.27227 | 0.357877 | 2.09211 | 0.216048 | 1.02787 |
| Ketoisoleucine | Plasma | 0.43903 | 2.26104 | 0.124966 | 0.597911 | 0.566073 | 2.20421 |
| hsCRP | Systemic | 0.350595 | 2.2061 | 0.280541 | 1.64002 | 0.056703 | 0.269769 |
| Fructose | Plasma | 0.412063 | 2.12216 | 0.38915 | 1.86192 | 0.198432 | 0.772666 |
| Number of total teeth | Oral | 0.326671 | 2.05556 | 0.516454 | 3.01914 | -0.03905 | 0.185767 |
| Lactate | Plasma | 0.377987 | 1.94666 | -0.00533 | 0.025498 | 0.590053 | 2.29759 |
| Allose | Plasma | 0.362576 | 1.8673 | 0.313492 | 1.49993 | -0.06041 | 0.235229 |
| HbA1c | Systemic | 0.294282 | 1.85176 | 0.234408 | 1.37033 | -0.05172 | 0.246072 |
| Uric acid | Plasma | 0.355429 | 1.83049 | 0.052759 | 0.252429 | 0.531137 | 2.06817 |
| BMI | Systemic | 0.284083 | 1.78758 | 0.142738 | 0.834435 | 0.330663 | 1.57316 |
| Pyruvate | Plasma | 0.346841 | 1.78626 | 0.270235 | 1.29297 | 0.297192 | 1.15722 |
| β-Alanine | Plasma | 0.341508 | 1.75879 | 0.145401 | 0.695683 | 0.44522 | 1.73363 |
| Cadaverine | Saliva | 0.390052 | 1.72634 | 0.353965 | 1.45544 | 0.326144 | 1.09139 |
| Isoleucine | Plasma | 0.328974 | 1.69424 | -0.04654 | 0.222677 | 0.551693 | 2.14822 |
| Acetoacetate | Systemic | 0.267939 | 1.68599 | 0.221803 | 1.29664 | -0.40772 | 1.93975 |
| Oleic acid | Saliva | 0.367778 | 1.62776 | 0.388147 | 1.59599 | 0.305376 | 1.02189 |
| 2-Aminobutyrate | Plasma | 0.312331 | 1.60853 | 0.394662 | 1.8883 | 0.117142 | 0.456137 |
| Mannose | Plasma | 0.311922 | 1.60642 | 0.36587 | 1.75054 | -0.38629 | 1.50415 |
| Number of decayed teeth | Oral | 0.253852 | 1.59735 | 0.185698 | 1.08557 | 0.226241 | 1.07636 |
| PlI | Oral | 0.246189 | 1.54913 | 0.251055 | 1.46764 | 0.26708 | 1.27066 |
| Threonate | Saliva | 0.343737 | 1.52135 | 0.336788 | 1.38481 | 0.537556 | 1.79885 |
| Creatinine | Systemic | 0.238663 | 1.50177 | -0.04653 | 0.272029 | 0.542554 | 2.58125 |
| Fasting plasma glucose | Systemic | 0.231246 | 1.4551 | 0.243136 | 1.42135 | -0.25353 | 1.20619 |
| Number of sound teeth | Oral | 0.231124 | 1.45434 | 0.238659 | 1.39518 | 0.200138 | 0.952174 |
| Tyrosine | Plasma | 0.278898 | 1.43635 | 0.0327 | 0.156458 | 0.541761 | 2.10954 |
| Waist circumference | Systemic | 0.226436 | 1.42484 | 0.161336 | 0.943157 | 0.286648 | 1.36376 |
| 2-Hydroxybutyrate | Plasma | 0.275798 | 1.42038 | 0.12239 | 0.585585 | 0.159915 | 0.622688 |
| 3-Hydroxybutyrate | Systemic | 0.222773 | 1.40179 | 0.319813 | 1.86959 | -0.52221 | 2.48446 |
| Urine albumin | Systemic | 0.217958 | 1.37149 | 0.354135 | 2.07024 | 0.056025 | 0.266543 |
| Glycated albumin | Systemic | 0.209735 | 1.31975 | 0.07951 | 0.464808 | -0.29192 | 1.38883 |
| Urine creatinine | Systemic | 0.20096 | 1.26453 | 0.061506 | 0.359557 | 0.194288 | 0.924342 |
| Leucine | Plasma | 0.238241 | 1.22696 | -0.09847 | 0.47113 | 0.503039 | 1.95876 |
| Indoleacetaldehyde | Plasma | 0.235441 | 1.21254 | 0.203552 | 0.973914 | -0.04746 | 0.18479 |
| Hydrocinnamate | Saliva | 0.267628 | 1.1845 | 0.157737 | 0.648588 | 0.401346 | 1.34304 |
| Galactose | Plasma | 0.228558 | 1.17709 | 0.181656 | 0.869148 | -0.14693 | 0.572135 |
| 3-Hydroxybutyrate | Plasma | 0.223271 | 1.14986 | 0.304978 | 1.45919 | -0.49517 | 1.92813 |
| *N*-Acetylputrescine | Saliva | 0.256164 | 1.13376 | 0.282585 | 1.16194 | -0.05463 | 0.182821 |
| Malate | Saliva | 0.252107 | 1.1158 | 0.415115 | 1.70688 | 0.142681 | 0.477459 |
| Tryptophan | Plasma | 0.204378 | 1.05256 | -0.07572 | 0.362309 | 0.57625 | 2.24384 |
| Putrescine | Saliva | -0.2271 | 1.00513 | -0.0962 | 0.395575 | 0.037396 | 0.12514 |
| Elaidic acid | Plasma | -0.19726 | 1.0159 | -0.09731 | 0.465572 | -0.45522 | 1.77257 |
| Lauric acid | Plasma | -0.19754 | 1.01736 | -0.28047 | 1.34195 | -0.12007 | 0.467543 |
| Phenylalanine | Saliva | -0.23165 | 1.02528 | 0.106902 | 0.439561 | -0.03808 | 0.127434 |
| Proline | Saliva | -0.2406 | 1.06486 | -0.06062 | 0.24924 | 0.006937 | 0.023214 |
| Serine | Plasma | -0.20701 | 1.06614 | -0.29159 | 1.39513 | -0.05931 | 0.230939 |
| Hypotaurine | Plasma | -0.20705 | 1.06632 | -0.24992 | 1.19577 | 0.01014 | 0.039484 |
| Isoleucine | Saliva | -0.24886 | 1.10142 | 0.070143 | 0.288415 | 0.013787 | 0.046138 |
| Pyroglutamate | Saliva | -0.25366 | 1.12266 | 0.016247 | 0.066806 | -0.03356 | 0.112291 |
| Citrate | Plasma | -0.21881 | 1.12689 | -0.3333 | 1.59469 | -0.35335 | 1.37588 |
| Oleic acid | Plasma | -0.22147 | 1.1406 | -0.11908 | 0.569732 | -0.55506 | 2.16132 |
| Hydroxyproline | Saliva | -0.26052 | 1.15302 | -0.1615 | 0.664055 | 0.022753 | 0.076141 |
| Phosphate | Plasma | -0.22438 | 1.15557 | -0.58687 | 2.80792 | 0.010741 | 0.041823 |
| Glutamine | Plasma | -0.22657 | 1.16687 | -0.40638 | 1.94434 | 0.153878 | 0.599179 |
| Urocanate | Saliva | -0.265 | 1.17287 | -0.17393 | 0.715183 | 0.033469 | 0.111997 |
| Dihydrouracil | Saliva | -0.27451 | 1.21496 | -0.07758 | 0.318983 | -0.09581 | 0.320607 |
| Age | Systemic | -0.20424 | 1.28515 | -0.32507 | 1.90034 | -0.22723 | 1.08105 |
| β-Glutamic acid | Saliva | -0.2966 | 1.31275 | -0.26769 | 1.10067 | -0.11671 | 0.39055 |
| Glutamine | Saliva | -0.30037 | 1.32942 | -0.03069 | 0.126188 | -0.07196 | 0.24079 |
| Number of treated teeth | Oral | -0.21207 | 1.33443 | 0.084352 | 0.493115 | -0.45221 | 2.15142 |
| Phosphate | Saliva | -0.30793 | 1.36285 | -0.31752 | 1.30557 | -0.48555 | 1.62483 |
| Glucuronate | Plasma | -0.2752 | 1.41731 | -0.30886 | 1.47775 | -0.2955 | 1.15064 |
| Pentadecanoic acid | Plasma | -0.28867 | 1.48668 | -0.23829 | 1.1401 | -0.55153 | 2.1476 |
| Linoleic acid | Plasma | -0.30989 | 1.59595 | -0.11968 | 0.572638 | -0.53223 | 2.07241 |
| HDL-C | Systemic | -0.30627 | 1.92721 | -0.14125 | 0.825744 | -0.39792 | 1.89313 |
| Myristic acid | Plasma | -0.39927 | 2.05628 | -0.38553 | 1.84461 | -0.38893 | 1.51442 |
| Number of missing teeth | Oral | -0.33432 | 2.10369 | -0.51645 | 3.01914 | 0.033768 | 0.160656 |

ALT, Alanine aminotransferase: AST, Aspartate aminotransferase; BMI, Body mass index; FDR, False discovery rate; HDL-C, High-density lipoprotein cholesterol; hsCRP, High-sensitivity C-reactive protein; OPLS, Orthogonal partial least square; Oral, Oral clinical indices; Plasma, Plasma metabolites; Saliva, Salivary metabolites; Systemic, Systemic clinical indices; PESA, Periodontal epithelial surface area; PISA, Periodontal inflamed surface area; VIP pred, Variable importance in projection on predictive component.
